# The chemical profiles and cytotoxicity of gaharu bouya oil from Borneo’s *Gonystylus bancanus* wood

**DOI:** 10.1038/s41598-024-58529-2

**Published:** 2024-05-27

**Authors:** Ika Oktavianawati, Mardi Santoso, Sri Fatmawati

**Affiliations:** 1 Department of Chemistry, Faculty of Science and Data Analytics, Institut Teknologi Sepuluh Nopember, Kampus ITS, Sukolilo, Surabaya, 60111 Indonesia; 2 Department of Chemistry, Faculty of Mathematic and Sciences, Universitas Jember, Kampus Tegalboto, Jember, 68121 Indonesia; 3 Agrifood and Biotechnology Research Center, ITS, Surabaya, Indonesia

**Keywords:** Chemical biology, Chemistry

## Abstract

Gaharu bouya oil obtained from distillation of the woods from *Gonystylus* genus has attracted essential oil industry interest. However, the information about gaharu bouya essential oil profile is limited. The presence of *Gonystylus* species is also critically endangered on the IUCN Red List. Therefore, exploring the -omics profiles of *Gonystylus bancanus*, a native plant from Borneo Island, is important for Indonesia to conserve the population. This research investigated the metabolite profiling of *G. bancanus* oil, especially the volatile components of its essential oils. Distillations were performed in two technical ways: hydrodistillation on a laboratory scale and steam distillation on an industrial scale. According to LC–MS and GC–MS profiles, both essential oils displayed similar chemical compositions. This article also discusses the similarity of the chemical contents of gaharu bouya oil and agarwood oil from the gaharu superior type (*Aquilaria*) to support the value of the oil. This research also investigated the cytotoxicity of gaharu bouya oil against three cell lines: HeLa, MCF-7, and HT-29.

## Introduction

Gaharu bouya oil, which is commonly known as agarwood bouya; gaharu buaya or crocodile agarwood; and aetoxylon oil, is an essential oil distilled from ramin wood (*Gonystylus bancanus*) or zebra wood (*Aetoxylon sympetalum*)^1^. This essential oil has attracted much interest in the essential oil industry and become one main valuable exported products from Indonesia. The commercial name of essential oil from this ramin wood, gaharu bouya, was taken from its pleasant smell, similar to the aroma of gaharu or agarwood from *Aquilaria* species, but it lacks lemon aroma^2^. It is suspected that gaharu bouya oil contains some compounds similar to agarwood. Hence, gaharu bouya is often referred as an inferior type of gaharu or agarwood^3^.

Taxonomically, *Gonystylus* and *Aetoxylon* are in the same family as *Aquilaria* and *Gyrinops*, i.e., Thymeleaceae family, in producing fragrant woods^4–6^. However, the quality grade of the resins or essential oils from gaharu bouya or ramin wood is lower than the agarwood from Aquilaria genus. Hence, gaharu bouya oil is cheaper than agarwood oil at about $300 per kg from essential oil distillers or farmers. Gaharu bouya oils showed many functional benefits: for religious purposes, perfume ingredients, and medicinal properties such as increased appetite and relaxation^7–10^. On the other hand, some research showed negative health impacts on using or interacting with the wood or even the dust^11–13^.

This article discusses the metabolite profiling of gaharu bouya oil compared to superior gaharu or agarwood from *Aquilaria* genus. Hence, it also explains the increasing demand for exporting gaharu bouya oil in international trading. The scarcity issue of *G. bancanus*, as shown in Appendix II of the IUCN Red List, also becomes an essential aspect of conservation^14–16^. In fact, *G. bancanus* is a lowland peat swamp forest native species of Borneo Island, Sumatra, and Peninsular Malaysia. Therefore, research on *G. bancanus* must be explored by us to conserve this species. By performing an important issue on the benefit of gaharu bouya oil, it would stimulate the government to develop a policy related to the conservation of *G. bancanus*.

To our knowledge, little is known about analyzing gaharu bouya oil from *G. bancanus* or ramin wood^17^. Therefore, this study deals with profiling gaharu bouya oils produced by steam distillation and hydrodistillation. Further research on bioactivity assays of gaharu bouya oil against three cell lines has been investigated and presented in this paper.

## Results

### Species barcoding of the wood

**Figure 1:**
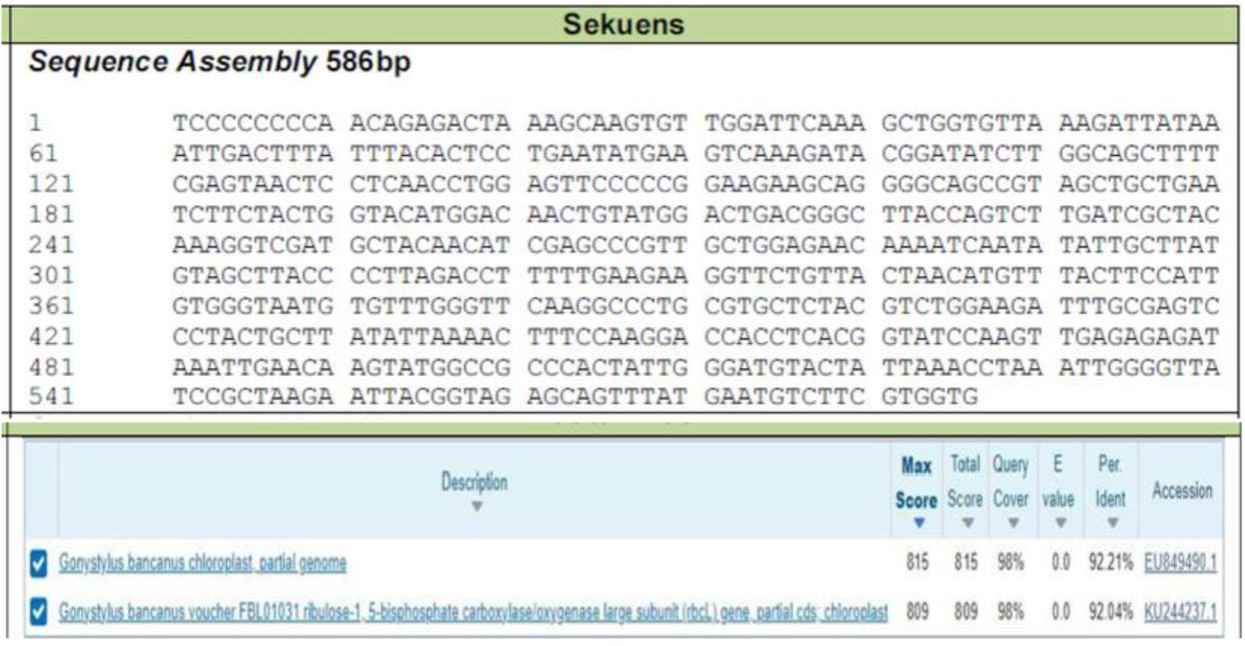
Sequence assembly (586 bp) result from the amplified product and the top two hit BLAST related to DNA target of *Gonystylus bancanus*.

The wood sample was identified for its taxonomy using the DNA barcoding method. Three steps for this species barcoding are genomic DNA extraction, PCR amplification, and bi-directional sequencing. The bioinformatic analysis results in sequence assembly and BLAST result as shown in Fig. 1.

### Characteristic of the wood

**Figure 2:**
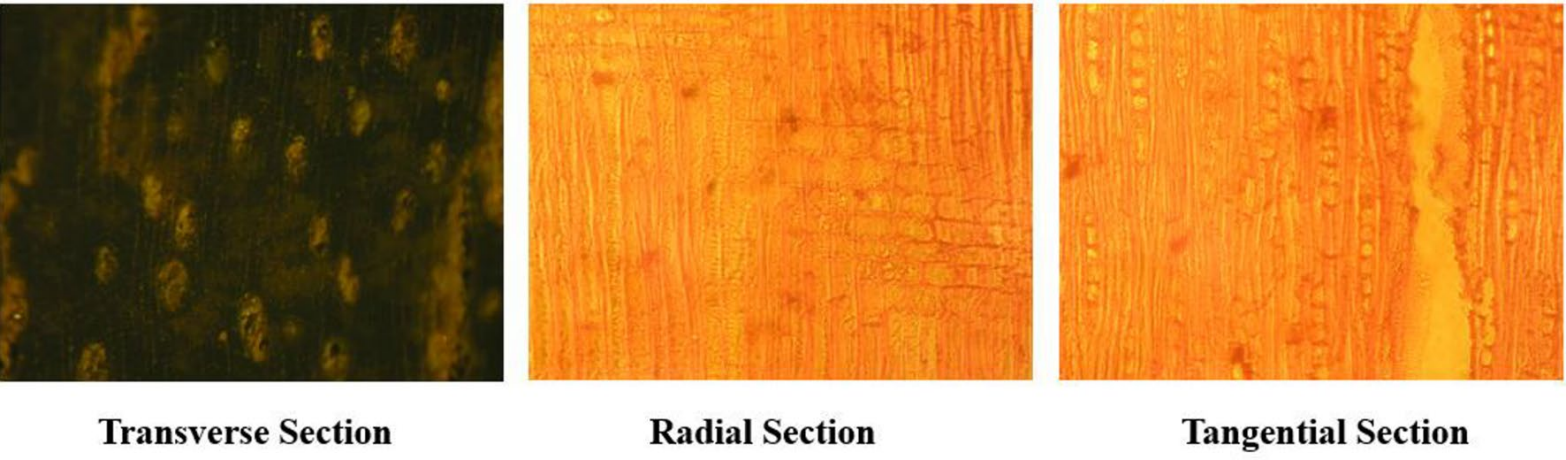
The illustration of ramin wood as *Gonystylus bancanus* is based on three sections: transverse (cross-section), radial, and tangential sections, observed under a light microscope.

**Figure 3:**
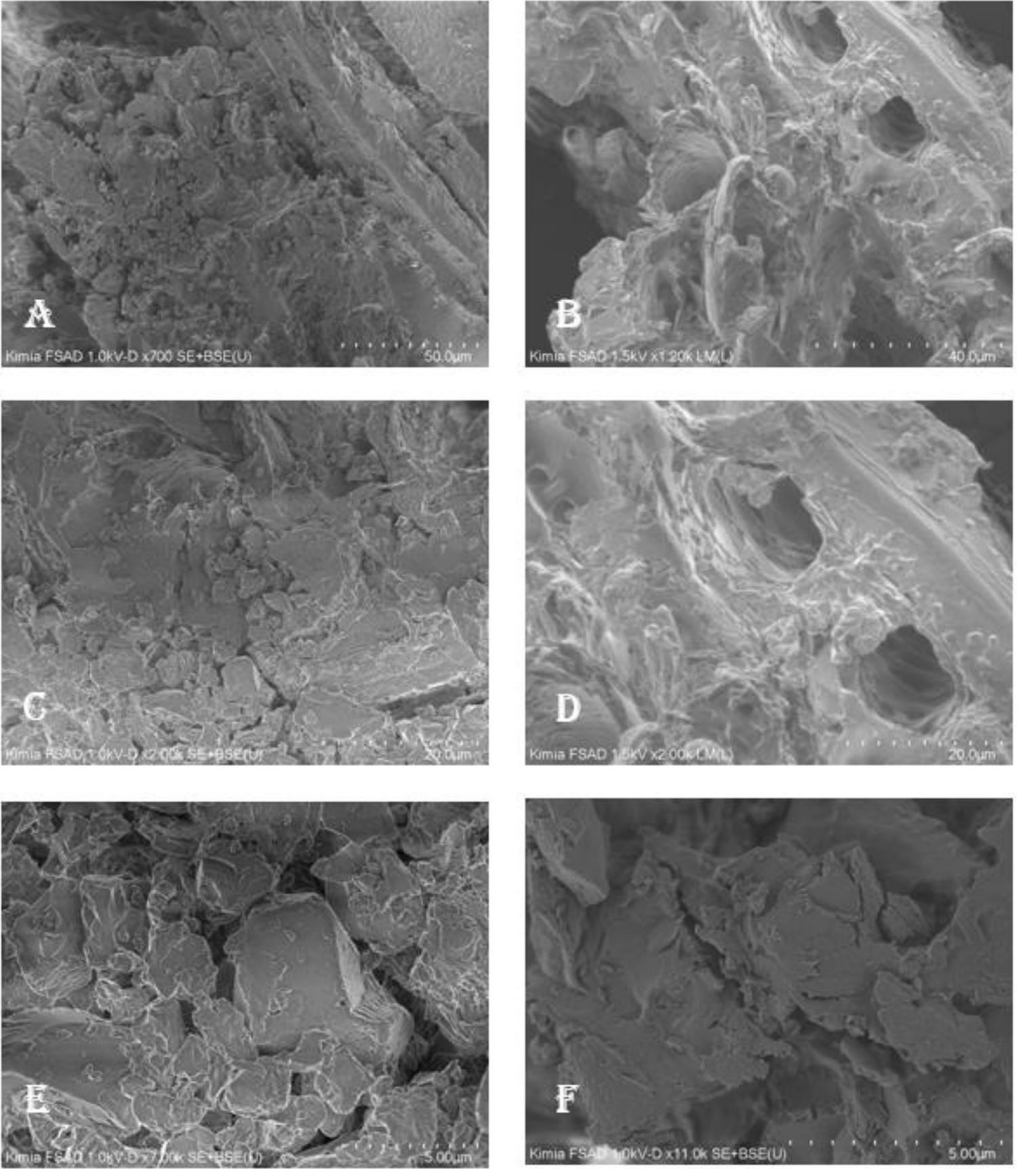
FESEM images of ramin wood powder before the treatment (panels on the left-hand side: (**A,C,E**) and after the treatment (panels on the right-hand side: (**B,D,F**) at × 700 (**A**); × 1.20k (**B**); × 2.00k (**C,D**); × 7.00k (**E**); and × 11.00k (**F**) magnifications.

The wood sample was obtained from *Gonystylus bancanus* as a part of *Thymelaeceae* family. The wood has a specific characteristic, as illustrated in Fig. 2, which was observed under the light microscope. The wood powder as the sample of essential oil extraction was also characterized for its topography using FESEM (Fig. 3).

### Chromatography analysis of the essential oils

**Figure 4:**
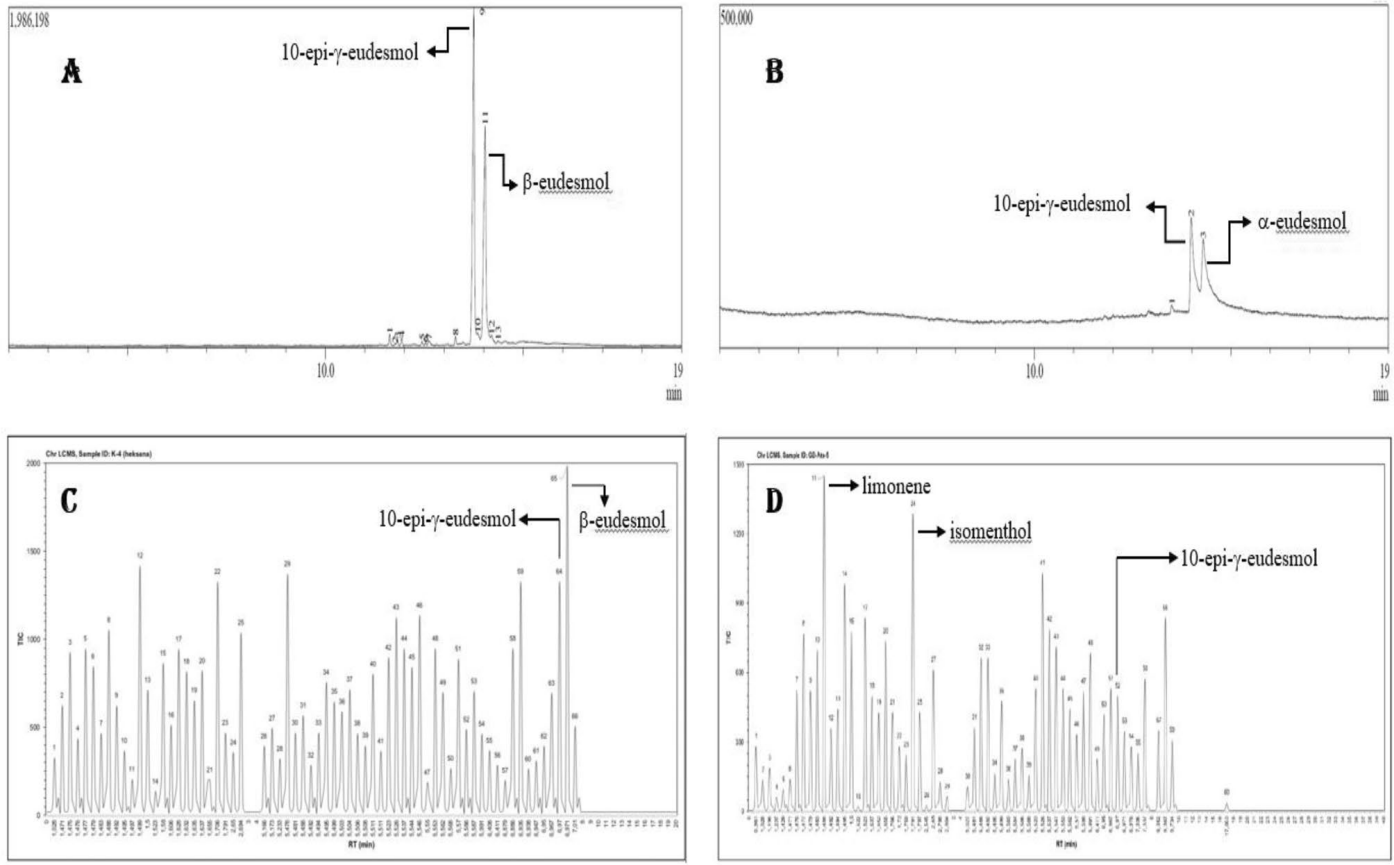
Chromatograms of GCMS (**A,B**) and LCMS (**C,D**) from gaharu bouya oils resulted from steam distillation (**A,C**) and hydrodistillation (**B,D**) processes.

**Figure 5:**
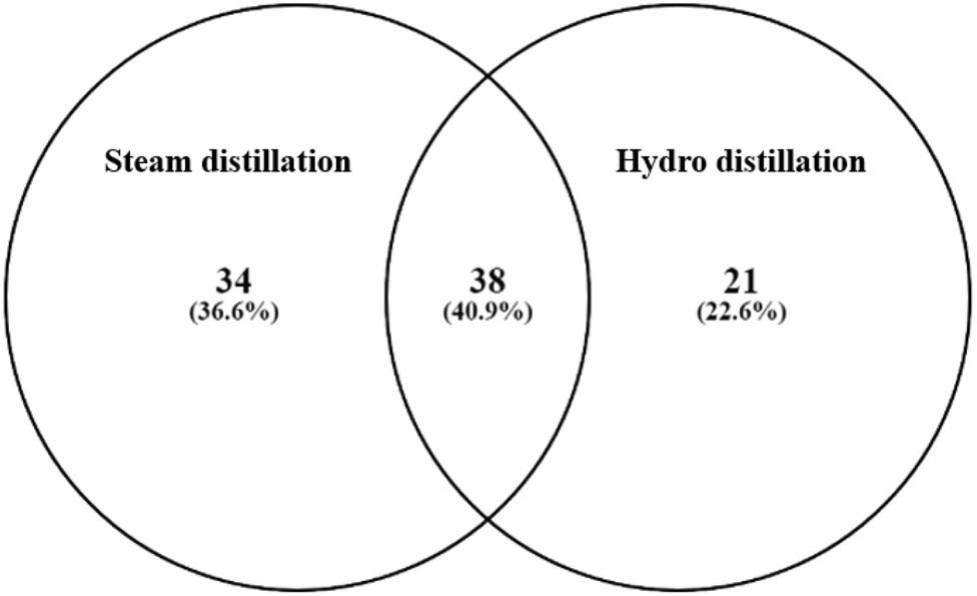
Venn diagram of identified compounds in the essential oils from steam distillation and hydrodistillation method obtained from the data of LCMS and GCMS at Table 1.

The gaharu bouya oils from hydrodistillation (current research) and steam distillation (commercial) processes were compared based on their chromatography profiles identified using GCMS and LCMS (Fig. 4, Table 1). Statistical analysis of those chromatography profiles was correlated and discriminated using multivariate data analysis PCA, as shown in Fig. 5.

**Table 1:** Chemical components in gaharu bouya oils analyzed by LCMS and GCMS.

Peak	Compounds	LCMS		GCMS	
		SD	HD	SD	HD
Terpenoids					
1	Isoprene	0.72	0.51	–	–
2	p-Cymene	1.38	0.53	–	–
3	β-Ocimene	2.04	–	–	–
4	α-Terpinene	0.96	1.95	–	–
5	α-Pinene	2.09	2.85	–	–
6	β-Pinene	1.87	1.94	–	–
7	Sabinene	1.03	2.59	–	–
8	Limonene	2.32	5.37	–	–
9	α-Terpinolene	1.38	1.34	–	–
10	β-Phellandrene	0.81	3.65	–	–
11	Camphene	0.45	–	–	–
12	α-Thujene	3.13	–	–	–
13	β-Myrcene	1.57	2.88	–	–
14	γ-Terpinene	0.30	3.10	–	–
15	1,8-Cineole	2.08	–	–	–
16	Borneol	1.44	–	–	–
17	Linalool	1.81	1.85	–	–
18	Isoborneol	–	1.59	–	–
19	cis-rose oxide	0.44	2.73	–	–
20	Geranial	2.93	1.59	–	–
21	p-Menthadiene	–	0.91	–	–
22	Isomenthol	1.03	4.76	–	–
23	Neoisomenthol	–	1.60	–	–
24	Borneol acetate	0.87	–	–	–
25	Linalyl acetate	1.10	–	–	–
26	α-Calacorene	0.71	–	–	–
27	Seychellene	3.02	–	–	–
28	α-Cubebene	1.03	1.35	–	–
29	α-Copaene	1.25	2.46	–	–
30	Longifolene	0.63	2.46	–	–
31	β-Caryophyllene	1.04	–	0.20	–
32	β-Chamigrene	1.12	0.61	–	–
33	β-Selinene	1.42	1.77	0.59	2.59
34	β-Bisabolene	1.30	0.52	–	–
35	β-Elemene	1.58	0.85	–	–
36	α-Humulene	1.02	1.02	0.79	–
37	α-Muurolene	0.87	0.59	–	–
38	Cycloseychellene	1.77	–	–	–
39	α-Bulnesene	0.81	–	–	–
40	(E,E)-α-farnesene	1.98	1.97	–	–
41	α-Bourbonene	2.48	3.82	–	–
42	Allo-aromadendrene	2.09	2.92	0.97	–
43	γ-Cadinene	1.86	–	–	–
44	Valencene	2.51	–	–	–
45	α-Cadinene	–	2.64	–	–
46	γ-Caryophyllene	0.41	–	–	–
47	α-Ylangene	2.09	1.97	–	–
48	Bicyclo-germacrene	1.54	1.65	–	–
49	Viridiflorene	0.59	-	–	–
50	γ-Cadinene	1.96	1.24	–	–
51	γ-Muurolene	–	1.93	–	–
52	Selina-4,11-diene	1.02	2.54	–	–
53	11-Selina-4-α-ol	0.63	0.85	–	–
54	Caryophylla-4(14),8(15)-dien-5α-ol	2.09	–	–	–
55	γ-Eudesmol	2.93	–	–	–
56	1-Epi-cubenol	0.58	–	–	–
57	α-Cadinol	0.68	–	–	–
58	Nerolidol	0.87	1.55	–	–
59	trans-farnesol	1.53	1.97	–	–
60	10-Epi-γ-eudesmol	2.93	1.86	49.92	58.88
61	β-Eudesmol	4.38	1.29	2.11	38.53
62	Elemol	–	1.05	0.67	–
63	Viridiflorol	1.12	–	–	–
64	9-β-Pimara-7,15-diene	–	1.30	–	–
65	Stemar-13-ene	–	3.10	–	–
66	Ent-cassa-12,15-diene	–	1.15	–	–
67	δ7-Avenasterol	–	0.15	–	–
68	α-Eudesmane	–	–	0.17	–
69	α-Selinene	–	–	0.75	–
70	α-Gurjunene	–	–	0.38	–
71	γ-Gurjunene	–	–	0.25	–
72	α-Eudesmol	–	–	42.78	–
73	4,4-Dimethyl-adamantan-2-ol	–	–	0.42	–
	Total of terpenoids	85.59	88.32	100.00	100.00
Aromatics (benzene-derivatives)					
1	Anethole	1.91	–	–	–
2	Safrole	0.79	2.27	–	–
3	Eugenol	2.29	–	–	–
4	Methyl salicylate	1.13	–	–	–
5	Isopentyl benzoate	–	0.40	–	–
6	Isoeugenitol	1.08	–	–	–
7	Eugenol acetate	1.56	–	–	–
8	Benzyl benzoate	0.81	–	–	–
9	Eugenetin	0.44	–	–	–
	Total of aromatics	10.01	2.67	0.00	0.00
Alkaloids					
1	Xanthine	–	0.10	–	–
2	7-Methyl xanthine	–	0.48	–	–
3	3-Methylxanthine	–	0.24	–	–
	Total alkaloids	–	0.82	–	–
Flavanoid					
1	3-Flavanol	–	0.94	–	–
	Total of flavonoid	–	0.94	–	–
Miscellaneous					
1	1,3-Pentadiene	–	1.05	–	–
2	2-Methylhexane	–	0.70	–	–
3	4-Heptenal	–	0.23	–	–
4	1,5-Octadien-3-one	–	0.35	–	–
5	1-Octen-3-ol	–	1.65	–	–
6	1-Octen-3-one	–	0.08	–	–
7	α-Champolenal	1.80	–	–	–
8	1-Butyl-2-propylcyclopentane	–	1.05	–	–
9	2-Hexyl-1-decanol	–	2.13	–	–
	Total of miscellaneous	1.80	7.24	–	–
		97.41	100.00	100.00	100.00

*SD* steam distillation, *HD* hydro distillation.

### Cytotoxicity evaluation of *Gonystylus bancanus* oil

**Figure 6:**
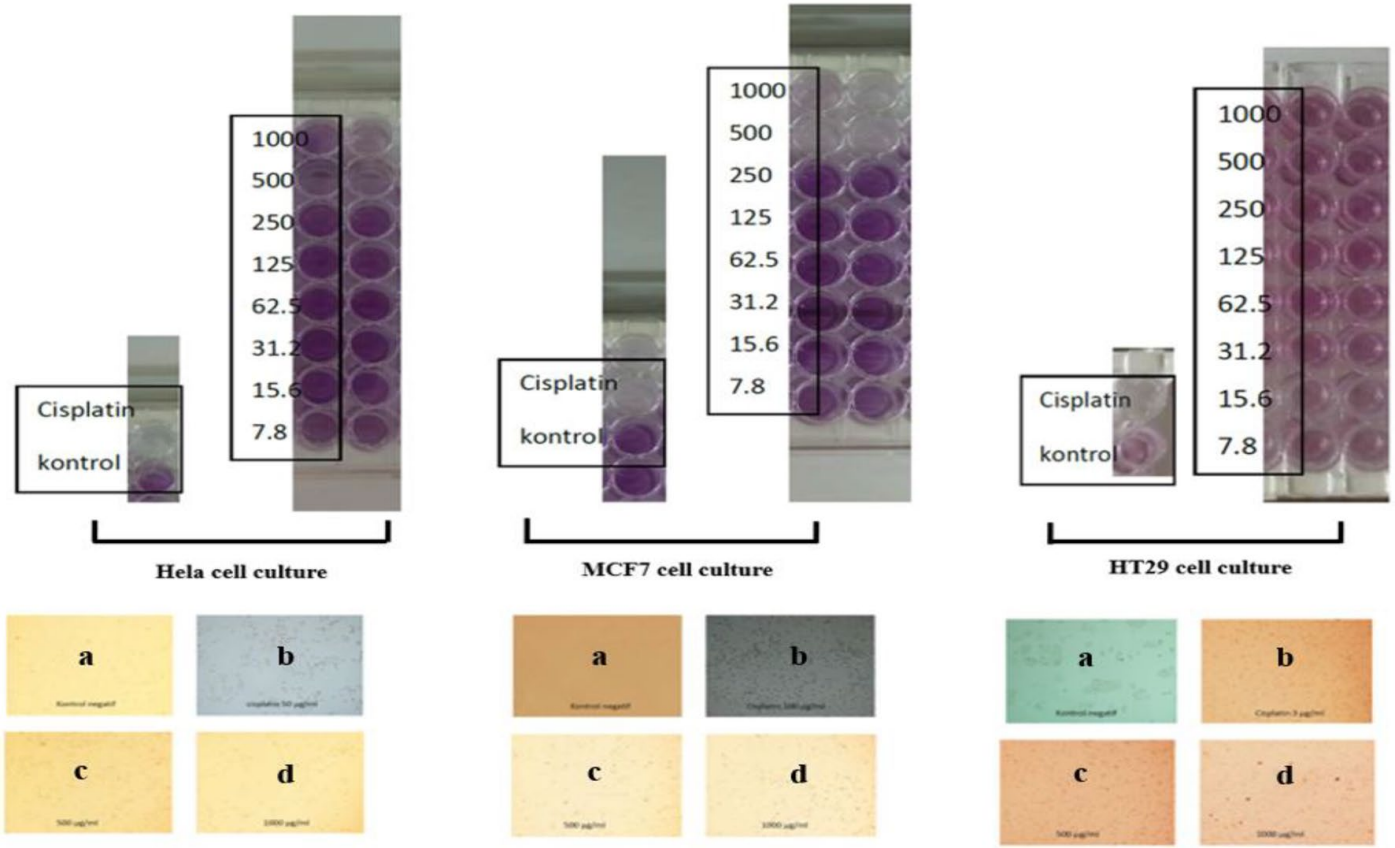
Cytotoxicities of *G. bancanus* essential oils (in the concentration of 7,8125; 15,625; 31,25; 62,5; 125; 250; 500; and 1000 μg/mL) against Hela, MCF7, and HT-29 cell cultures. *a*: negative control; *b*: cisplatin; and representatives of *G. bancanus* oils in *c*: 500 μg/mL and *d*: 1000 μg/mL.

Cytotoxic activities of gaharu bouya oil were screened on three cell lines: HeLa, MCF-7, and HT-29. The results were described in Fig. 6 and Table 2.

**Table 2:** Comparison of *G. bancanus* oil and positive control (cisplatin)’s concentrations at fifty percent inhibition to the culture cells: Hela, MCF7, and HT-29.

Samples	IC_50_ (μg/mL) ± stdv		
	Hela	MCF7	HT-29
*G. bancanus* oil	430.21 ± 0.09	102.34 ± 0.09	836.46 ± 0.03
Cisplatin	41.01	100.00	2.98

## Discussion

Ramin wood, identified as *Gonystylus bancanus*, is a kind of hardwood from the *Thymelaceae* family. All parts of the wood (including sapwood and heartwood), except the bark, were processed together as the sample of *G. bancanus* wood. This wood has been determined for its taxonomy using species barcoding from the DNA sequencing method, and the result is presented in Fig. 1. The BLAST on NCBI results in two hits of related target DNA sequences, i.e., *Gonystylus bancanus* chloroplast (partial genome) (https://www.ncbi.nlm.nih.gov/nuccore/EU849490.1) and ribulose-1,5-biphosphate carboxylase/oxygenase large subunit (rbcL) gene (https://www.ncbi.nlm.nih.gov/nuccore/KU244237.1) with the percentage identities of 92.21% and 92.04%, respectively.

The wood was also identified based on its wood anatomy with three slice forms of ramin wood in transversal, radial, and tangential sections, as seen in Fig. 2. The observation under the microscope on a slice of ramin wood showed that the wood grain (texture) had a straight direction and a smooth surface, with an oval-shaped porosity, and were mostly solitary cells. The parenchyma cells were axially paratracheal, with a thin wing, aliform, and some of them were short tangential bands, but no axial intercellular duct was found. It was assumed that gum conduction did not occur in this wood. This wood has a density of 0.60 g/cm^3^, which therefore was grouped in the wood with a strength of class III. A cross-section of the wood showed that the dark lines across this transverse section represented the cut of axial cells. A few dark wavy lines in the tangential longitudinal indicated the presence of latewood. Horizontal lines in the radial area of the wood represented the rays, while the vertical lines supported the fact that it was latewood, marked with densely-layered cells.

Microstructures of wood powder before and after the treatment were also observed using FESEM. Before the treatment, ramin wood powder appeared rough and hard, while after the treatment, the powder had less surface roughness, slightly smoother, and softer (Fig. 3E,F). The treatment created holes (Fig. 3B,D) due to the interaction of materials with hot water during distillation. These empty spaces were proposed to be formed mainly due to the loss of some chemical components or complex molecules extruding through the ramin wood surfaces. Physically, the treatment affected the topology, roughness, and chemical composition of materials, as shown by the images of the epidermic layer on the wood surfaces between the left and right sides in Fig. 3.

Distillation is a standard method for extracting essential oil from plant parts. Gaharu bouya oil produced by hydrodistillation resulted in a 0.90 ± 0.01% yield, while industrial scale using steam distillation resulted in an oil yield of up to 1%. Determining the total phenolic and flavonoid content of gaharu bouya essential oil afforded the values of 64.79 ± 1.58 mg GAE/g essential oil and 0.21 ± 0.07 mg QE/g essential oil, respectively.

Chromatography analysis of gaharu bouya oils was performed using GCMS and LCMS (Fig. 4). Chromatograms from GCMS data showed that both gaharu bouya oils exhibited a similar pattern of peaks for the compounds represented inside the oils. Both oils contained two significant peaks, which were not significantly different, but both mass spectral data predicted different compounds in detail. Two major peaks in GCMS data from commercial gaharu bouya oil represented 10-epi-γ-eudesmol and β-eudesmol, while from this research, gaharu bouya oil signified 10-epi-γ-eudesmol and α-eudesmol. However, LCMS data strengthen the results that eudesmol isomers, i.e., 10-epi-γ-eudesmol, β-eudesmol, and α-eudesmol, are similar in the significant amounts of the oils. The predicted compound, γ-eudesmol, also presented in the commercial gaharu bouya oil as an isomer of 10-epi-γ-eudesmol, hence the amounts of 10-epi-γ-eudesmol would be the total of both isomers of γ-eudesmol. The Venn diagram of chemical components in both essential oils from two distillation methods in Fig. 5 also shows an intersection as an overlap of 38 components in both oils, including two eudesmol isomers, i.e., β-eudesmol and 10-epi-γ-eudesmol. In the other hand, there are 34 components exclusively found in essential oil from steam distillation, and 21 other compounds are in hydrodistillation product.

**Figure 7:**
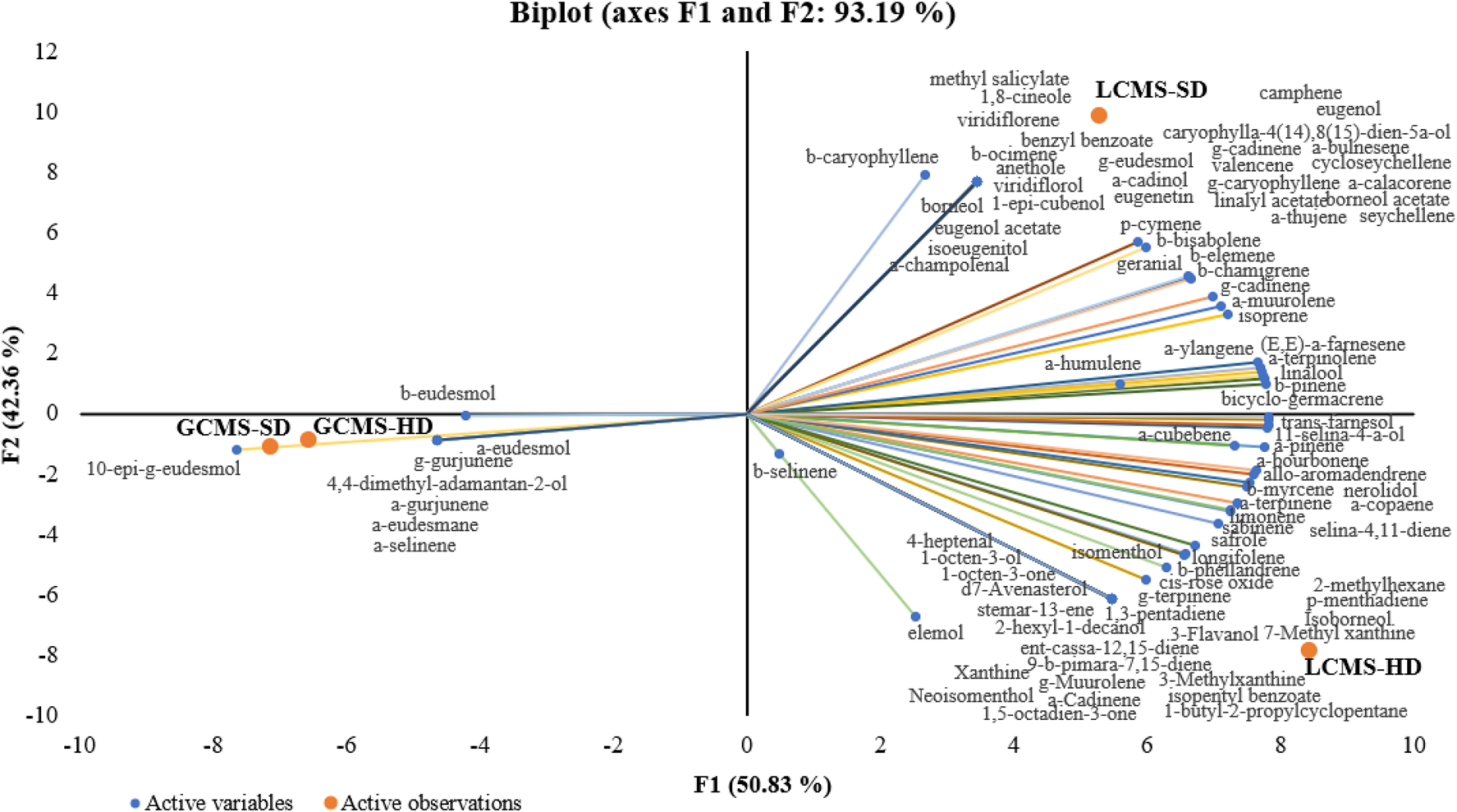
PCA biplot of LCMS and GCMS analysis for gaharu bouya oil from this research (*HD* hydrodistillation), and the commercial one (*SD* steam distillation).

Figure 7 shows that the analysis of LCMS and GCMS data (from Table 1) can be remarkably distinguished by PC1 (F1). The LCMS data resulted in various chemical components in the superior quadrant, while the GCMS data was in the negative quadrant of PC1. Interestingly, LCMS data of both samples (HD and SD) also resumed high variability of components separated by PC2 (F2). However, GCMS data of gaharu bouya oil from this research and the commercial one showed high similarity in chemical constituents. This PCA biplot implied that volatile compounds detected by GCMS analysis were similar in both samples (Table 1). It was also concluded that alcoholic sesquiterpenes with eudesmane skeleton, especially 10-epi-γ-eudesmol, β-eudesmol and α-eudesmol, which were the major volatile compounds, took more than 80% of oil compositions in gaharu bouya oil (Table 3).

**Table 3:** Major components in gaharu bouya oil based on LCMS and GCMS analysis.

Analysis	Steam Distillation		Hydrodistillation	
	Compounds	Abundance (%)	Compounds	Abundance (%)
LCMS	b-Eudesmol	4.38	Limonene	5.37
	a-Thujene	3.13	Isomenthol	4.76
	Seychellene	3.02	a-Bourbonene	3.82
	Geranial	2.93	b-Phellandrene	3.65
	10-Epi-g-eudesmol	2.93	g-Terpinene	3.10
GCMS	10-Epi-g-eudesmol	49.92	10-Epi-g-eudesmol	58.88
	a-Eudesmol	42.78	b-Eudesmol	38.53

**Figure 8:**
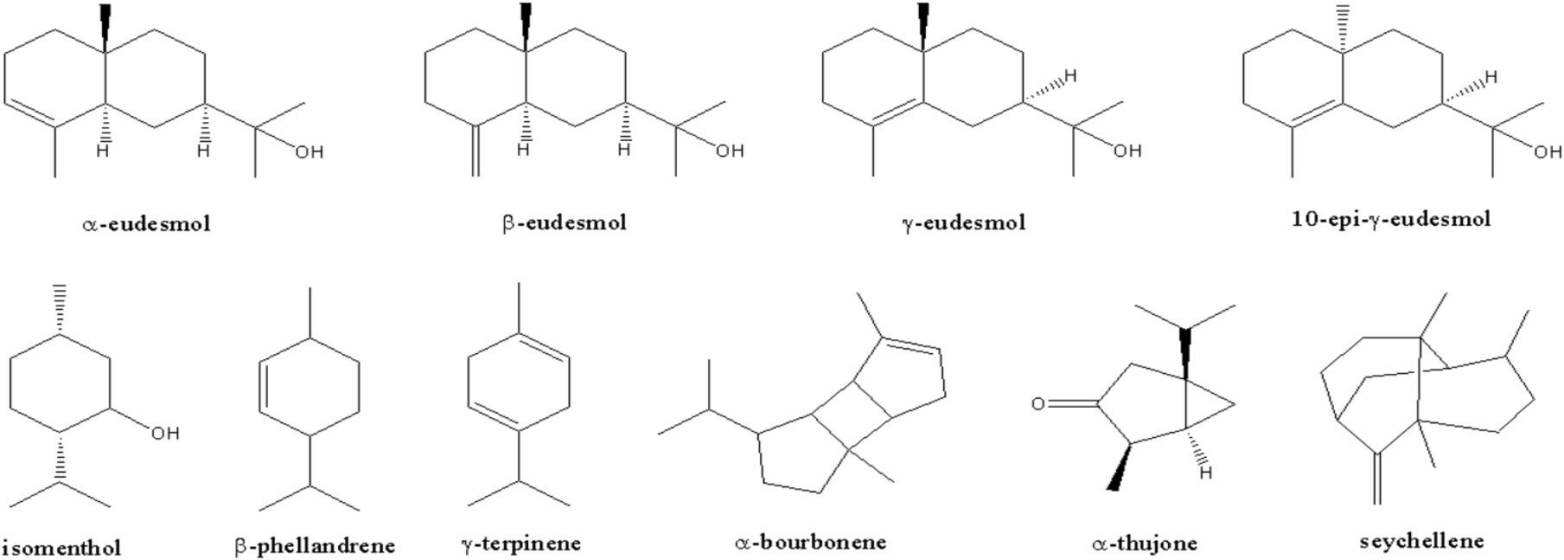
Structures of major compounds presented in gaharu bouya oil from *G. bancanus* wood.

As its name is so close to agarwood or gaharu, the gaharu bouya oil is assumed to contain similar components to agarwood from *Aquilaria* species. Some major chemical compounds in agarwood oil are eudesmane, nootkatone, cadinene, guaiane, prezizane, and agarospirane skeletons. Agarol, 4-nor-epi-γ-eudesmol, 10-epi-γ-eudesmol, selina-3,11-dien-14-ol, eudesm-4-ene-11,15-diol, and ent-4(15)-eudesmen-1α,11-diol are some compounds from agarwood having eudesmane skeletons^18^. Interestingly, our research result (Table 3, Fig. 8) showed that 10-epi-γ-eudesmol, as a marker compound of agarwood from Indonesian *Aquilaria malaccensis*^19^, is also found in gaharu bouya oil in a significant quantity, more than 50% of the oil.

According to that, although both distillation methods share 38 common components in their essential oils, including 10-epi-g-eudesmol, but the total components responsible for the fragrant odor, particularly for terpenoids (the sesquiterpenes) and benzene derivatives, are shown to be higher in the steam distillation product than in hydrodistillation. The physical performance of the essential oil from steam distillation appears transparent, viscous, and no solid form at fridge temperature (4 °C). In contrast, the oil from hydrodistillation forms a white solid lipid at low temperatures, which liquefies when the temperature rises. The oil yields obtained from both distillations showed that steam distillation produced a 10% higher oil yield than hydrodistillation. Therefore, it is suggested that steam distillation is a preferred method to be applied on the industrial scale of essential oil production since it produces higher oil yield, better physical performance, and metabolically satisfactory eudesmol content than hydrodistillation.

β-eudesmol, another eudesmol available in gaharu bouya, has been known to show a wide range of bioactivities in previous research^20^. Antimutagenic activity by suppressing *umu* gene expression 48% has been reached by applying β-eudesmol at less than 0.18 μmol/mL^21^. 10–100 μM of β-eudesmol inhibited proliferation of HeLa, SGC-7901, and BEL-7402^22^. It is also potential as an anti-cholangiocarcinoma candidate with IC_50_ value of 47.62 ± 9.54 μM at 24 h interaction against KKU-100 cells^23^. It inhibited the growth of HL-60 cells with an IC_50_ value of 35.1 μM^24^ and induced DNA fragmentation in HL-60 cells at 80 μM^25^. Remarkably, 950 ng of β-eudesmol intake could affect mental stress in humans^26^.

According to those references, cytotoxicity assays against some cell lines were carried out using gaharu bouya oil containing some eudesmol derivatives: β-eudesmol, 10-epi-γ-eudesmol, and α-eudesmol. Three cell lines, including HeLa, MCF7, and HT-29 have been used to represent cervical cancer, breast cancer, and human colon cancer cells, respectively (Fig. 6). Unfortunately, the cytotoxicity assay results for *G.bancanus* wood essential oils against those three cell lines were not in accordance with other research results on bioassay of β-eudesmol^22–25^. The IC_50_ values of gaharu bouya oils took about 10–400 times more than cisplatin, the standard chemotherapy medication (Table 2). It showed that only the MCF-7 cell line was inhibited in a similar concentration to cisplatin. However, the relatively high value reaches more than 100 μg/mL. Consequently, using gaharu bouya oil as a chemotherapeutic agent for several diseases including cervix, breast, and human colon cancer is impossible. However, the isolation of β-eudesmol and two other eudesmols: 10-epi-γ-eudesmol and α-eudesmol, as three major volatile compounds in gaharu bouya oil would be an essential step to conduct, followed by the analysis of its bioactivities against some cancer cell lines stated before.

## Conclusion

Gaharu bouya oil from *Gonystylus bancanus* contained mostly volatile components from terpenoids and benzene aromatics groups. The highest proportion component of gaharu bouya oil is 10-epi-γ-eudesmol, a marker compound found in gaharu superior from *Aquilaria malaccensis*. However, steam ditillation gains more advantageous for being applied in industrial scale since it produces higher oil yield, better oil physical performance, and comprehensive metabolomic than hydrodistillation. This fact broadens the benefit of gaharu bouya oil production from ramin wood since it can be beneficial as an additive or substitute for agarwood oil. Although cytotoxicity assays of gaharu bouya oil against HeLa and HT-29 did not positively impact inhibition, they showed comparable activity to cisplatin as the standard against MCF-7 cells. Therefore, gaharu bouya oil has a potency to be explored further, especially in isolating its chemical components, and to be investigated for its bioactivity as a candidate for an anti-breast cancer agent.

## Methods

### Plant materials

**Figure 9:**
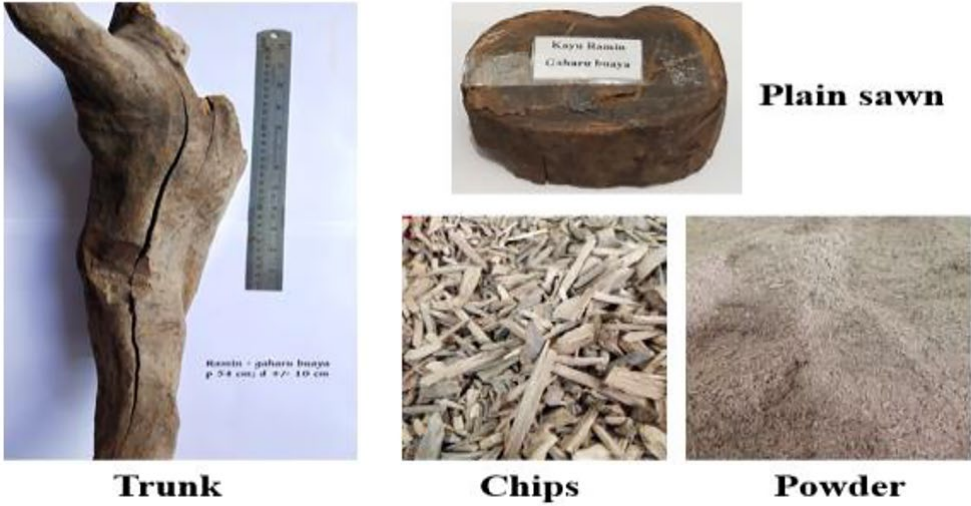
The ramin wood as part of *Gonystylus bancanus*.

The wood of *Gonystylus bancanus* (ramin) was initially obtained from the Kalimantan, Indonesia forest. This wood is not taken from its natural habitat by logging but from the wood parts that have fallen. Ramin wood has been marketed into Java as a legal log. The current sample of this research was obtained from the manufacturer and exporter of essential oils, PT Padaelo Sejahtera, Magelang, Central Java, Indonesia, which distributed the wood chips legitimately. This ramin wood (Fig. 9) was identified by species barcoding method from PT. Genetika Science Indonesia, and by wood anatomists Prof. Agus Sulistiyo Budi and Sri Wahyuni. This wood was acknowledged as a part of *Gonystylus bancanus*, and was deposited at the Laboratory of Biology and Wood Preservation, Faculty of Forestry, Universitas Mulawarman, Indonesia, under voucher number 220622-3. The supporting data for this identification was presented in the results and discussion section. The wood chips of ramin were ground into powder using a miller processor. This powder was subjected to further distillation to produce gaharu bouya oil. The commercial gaharu bouya oil was obtained from steam distillation of the same ramin wood provided by PT. Padaelo Sejahtera. The experimental field study adheres to pertinent institutional policies and conforms with pertinent laws.

### Methods

The methodology of this research consisted of six parts: (1) species barcoding of wood material; (2) characterization of wood chip materials; (3) isolation of essential oil using hydrodistillation; (4) total phenolic and flavonoid content; (5) chromatography analysis for chemical components identification, including LCMS and GCMS; (6) statistical analysis based on Principal Component Analysis (PCA); (7) cytotoxicity assays against three cell lines (HeLa, MCF7, and HT-29).

#### Species barcoding of wood materials

The wood sample was extracted for its DNA with Quick-DNA Plant/Seed Miniprep Kit (Zymo Research, D6020). PCR amplification was carried out using My Taq HS Red Mix kit, 2X (Bioline, BIO-25048). The electrophoresis of the amplified product was conducted on agarose gel 1% in TBE buffer based on rbcL gen. Bi-directional sequencing was applied based on Sanger DNA Sequencing by using Capillary Electrophoresis to produce a bioinformatic analysis result related to BLAST against NCBI database.

#### Characterization of wood materials

Characterization of wood materials as samples in this research included surface analysis of the slicing wood using a light microscope (Olympus BH2) and microstructure analysis of wood powder using FESEM Hitachi type Regulus 8220. FESEM (Field Emission Scanning Electron Microscopy) sample imaging was obtained from samples prepared as gold-coated specimens and imaged under high vacuum at 1.0 kV and 1.5 kV. SEM images were recorded at magnifications ranging from × 250 to × 11.00.

#### Distillation of ramin wood

Five hundred grams of wood powder was distilled using a Clevenger hydrodistillation set-up with an addition of 3.5 L aquadest. Distillation was run for 6 h and was counted for the first second of distillation at the first drop of distillate. The essential oil in the distillate was separated from the water and was dried using anhydrous magnesium sulfate. Resinous oil was obtained and kept at 5 °C until it was sent for further analysis using GCMS, LCMS, and bioassay.

Another sample of essential oil used in this research was obtained privately from an essential oil distiller, PT. Padaelo Sejahtera, with purity of 100% without any solvent addition. This gaharu bouya oil was extracted on an industrial scale using steam distillation for 36 h. Further analysis of this oil was delivered using LCMS and GCMS.

#### Total phenolic and flavonoid contents

Total phenolic content was determined based on the regular method applied in our laboratory^27,28^. The Modified Folin-Ciocalteu method was employed by mixing the sample (1000 ppm) with 10% Folin-Ciocalteu solution and 5% sodium carbonate. The total phenolic content was measured as absorbance at 765 nm to compare with gallic acid, as standard, using spectrophotometer Genesys, Thermo Fisher Scientific, Madison, WI, USA. This number was also expressed as a mg GAE (gallic acid equivalent) per gram of essential oil.

Total flavonoid content was evaluated using the modified Aluminium Chloride method as reported in the previous articles^27,29^. Briefly, the sample was mixed with 2% AlCl_3_ in methanol and incubated for 1 h at room temperature. The mixture was measured at 415 nm using a UV–Vis spectrophotometer. The absorbance of the sample was compared to a standard curve of quercetin. Total flavonoid content was calculated as mg QE (quercetin equivalent) per gram of essential oil.

#### Chromatography analysis

Liquid Chromatography Mass Spectrometry (LCMS) analysis was performed on a Shimadzu LCMS-8040 LC/MS equipped with a Shimadzu Shim Pack FC-ODS column of 2 mm × 150 mm, and 3 µm with mobile phase mode was isocratic with a flow rate of 0.5 mL/min and a sampling cone of 23.0 V. The MS-focused ion mode was ion type [M]^+^ with a collision energy of 5.0 V, desolvation gas flow of 60 mL/h, and desolvation temperature of 350 °C. The fragmentation method was low energy CID with ionization by ESI, scanning rate was 0.6 s/scan (m/z: 10–1500), source temperature was 100 °C, and run time was 80 min.

Gas chromatography–mass spectrometry (GCMS) analysis was run on Shimadzu GCMS-QP2010S equipped with DB-5MS column in 30 m length, 0.25 mm diameter, and 0.25 μm wide of a film with the column oven temperature was 70 °C held in 5 min, while injection temperature was 300 °C for 19 min. The ion source temperature for MS was 250 °C, the interface temperature was 305 °C with a solvent cut time of 3 min, and the detector gain mode was relatively + 0.00 kV.

Spectrums and their fragmentations obtained from LCMS and GCMS analysis were matched to the spectrum references under Mass Spectral Library of NIST20 and WILEY229—NIST62 databases, respectively. The instruments are regularly standardized using a reference mass of perfluorotributylamine (PFTBA, C_12_F_27_N) and PEG-PPG-Raffinose, respectively. These databases confirm a range of volatile and non-volatile compounds.

#### Statistical analysis

The diagnostic tool for statistical analysis in this research included a score plot and loading plot (shown in the biplot figures) of Principal Component Analysis (PCA), a dendrogram of Hierarchical Clustering Analysis (HCA), and a 3D plot of Origin software. A biplot analysis was performed on data from LCMS and GCMS analysis as a graph of extraction solvents toward the relative amount of correlated identified compounds. The clustering of the extracts was determined from the identified compounds resulting from the variation of solvent extraction and was mapped on HCA. Whenever the accumulative eigenvalue of PCs was less than 80.0%, an increasing matrix was compulsory to apply until the minimum PC value of 80.0% was obtained.

#### Cytotoxicity assay

Anticancer properties were identified by testing gaharu bouya oils against three cell lines: HeLa, MCF7, and HT-29. Cytotoxicity assay was performed based on the MTT method using CellQuanti-MTT™ Cell Viability Assay Kit (Cat. No. CQMT-500). The protocol is available on the BioAssay Systems website^30^. Anti-proliferative effects of those cell lines were observed using an inverted microscope and Elisa reader.

## Data Availability

All data generated and analyzed during this study are included in this paper. Further detail for those related data is available from the corresponding author on reasonable request.
